# miR-143-3p inhibits the proliferation, migration and invasion in osteosarcoma by targeting FOSL2

**DOI:** 10.1038/s41598-017-18739-3

**Published:** 2018-01-12

**Authors:** Xiangran Sun, Guo Dai, Ling Yu, Qingzhu Hu, Jingteng Chen, Weichun Guo

**Affiliations:** Department of Orthopedics, Renmin Hospital of Wuhan University, Wuhan, 430060 Hubei Province P. R. China

## Abstract

Osteosarcoma (OS) is the most common type of primary malignant bone tumor and mainly occurs in children and adolescent. Because of its early migration and invasion, OS has a poor prognosis. It has been reported that mircoRNAs (miRNAs) play a crucial role in the occurrence and development of multiple tumors. In this study, we identified the aberrant-expression of miR-143-3p in osteosarcoma and examined the role of miR-143-3p in OS development. Further, we searched the miR-143-3p target gene and verified its accuracy by luciferase experiments. Finally, we explored the relationship between miR-143-3p and FOS-Like antigen 2 (FOSL2). Our data indicated that miR-143-3p expression was substantially lower in OS tissues and cell-line compared with normal tissues, and was lower in patients with poor prognosis. In addition miR-143-3p inhibited OS cell proliferation and metastasis while promoting apoptosis. We next showed that FOSL2 was directly targeted by miR-143-3p and could reverse the inhibition caused by miR-143-3p. Finally, we found FOSL2 expression in OS cells was significantly higher compared with normal cells and negatively correlated with miR-143-3p. Thus, miR-143-3p directly and negatively targets FOSL2 to affect OS characteristics. This provides a new target for the treatment of OS and deserves further study.

## Introduction

Osteosarcoma (OS) is a common primary malignant bone tumor occurring in adolescents and children especially in those under 20 years of age^1^. At present, the origin of OS is not clear. Some reports suggest that OS may originate from mesenchymal cells^2,3^. With its characteristic early metastasis, the prognosis of OS is usually poor. The tumor cells can break through the cortex and medullary cavity and transfer through the blood to other tissues, especially the lung^4,5^. A small number of tumor cells can transfer to the brain, prostate and other tissues and lead to death^6^. Although, in recent years, the 5-year-survival-rate of OS patients has shown some increase with the improvement of medical technology, the survival rate is still very low^3^. At present, the metastasis of OS is still the main factor affecting the prognosis of OS patients.

MicroRNA (miRNA) is a single-stranded non-coding RNA containing 22–24 nucleotides and exists on non-coding regions^7^. As a regulator of mRNA^8^, it regulates the entire process of cell proliferation, gene expression and even ontogenesis^9^. It has been reported that abnormal expression of miRNAs is found in a large number of human tumors including renal carcinoma, colon cancer, lung cancer, glioblastoma and gastric cancer^6,10,11^. Recent studies have revealed that many miRNAs are expressed abnormally in OS cells and play a role as oncogenes or anti-oncogenes in the occurrence and development of OS^12,13^.

In this study, we examined differences in miR-143-3p expression between and normal tissues and explored the biological function of miR143-3p in proliferation, apoptosis, migration and invasion in OS cell lines and tissues. At the same time, we investigated the relationship between miR-143-3p and its target gene FOS-like antigen 2 (FOSL2) in the regulation of OS.

## Results

### miR-143-3p is significantly downregulated in OS tissues and OS cell lines

**Figure 1:**
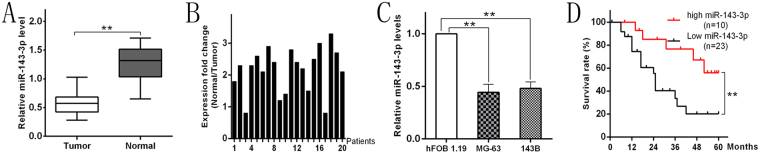
miR-143-3p downregulation in OS tissues and cell lines is associated with poor prognosis. **(A)** The miR-143-3p expression is significantly decreased in OS tissues. **(B)** miR-143-3p expression relative to adjacent normal tissues for 20 patients. **(C)** The miR-143-3p expression in MG-63 and 143B cell lines are significantly lower compare to hFOB 1.19 cell line determined by qRT-PCR. **(D)** Kaplan-Meier curves were performed for survival in all 33 patients.

We collected OS tissues and adjacent normal tissues from 20 tumor cases, and measured the expression of miR-143-3p in each specimen. qRT-PCR analysis showed that the expression of miR-143-3p was significantly lower in OS cells than in normal tissues (Fig. 1A). The ratio of miR-143-3p expression in normal tissue and tumor tissue was greater than 2 (N/T > 2) in 14 of 20 (70%) patients (Fig. 1B). At the same time, we analyzed the expression of miR-143-3p in three cell lines: MG-63, 143B and the normal human osteoblastic cell line hFOB 1.19, and found that the expression level of miR-143-3p in the human OS cell lines (MG-63, 143B) was significantly lower than that in normal human osteoblastic cells (Fig. 1C). The analysis of the relationship between miR-143-3p expression level and clinical features of OS patients is shown in Table 1.

**Table 1:** Correlation between miR-143-3p levels and clinicopathological variables of osteosarcoma patients.

Factor	Characteristics	Total Number (n = 33)	miR-143-3p expression		P-value
			Low level	High level	
Gender	Male	17	11	6	0.710
	Female	16	12	4	
Age (years)	<=20	23	15	7	0.840
	>20	10	7	3	
Anatomical site	Femure	17	12	5	0.891
	Tibia	10	8	2	
	Humerus	4	2	2	
	Others	2	1	1	
Clinical stage	II A-B	11	5	6	0.027^a^
	III A	16	13	3	
	III B	6	5	1	
Tumor size (cm)	<=5	13	6	7	0.026^a^
	>5	20	17	3	
Tumor grade	Low	18	16	2	0.019^a^
	High	15	7	8	

Note: ^a^Statistically significant P-value.

### miR-143-3p expression is lower in poor prognosis patients

To determine whether miR-143-3p expression correlates with clinical prognosis in patients, we gathered information of all 33 OS patients and used RT-PCR to measure the expression of miR-143-3p. The median value of miR-143-3p expression in all patients was used to divide the patients into two groups: a high expression group (>median, n = 10) and low expression group (<median, n = 23). From these statistical results, we found that the survival time of the high-expression group was longer compared to the low expression group (Fig. 1D).

### miR-143-3p inhibits proliferation of OS cells

**Figure 2:**
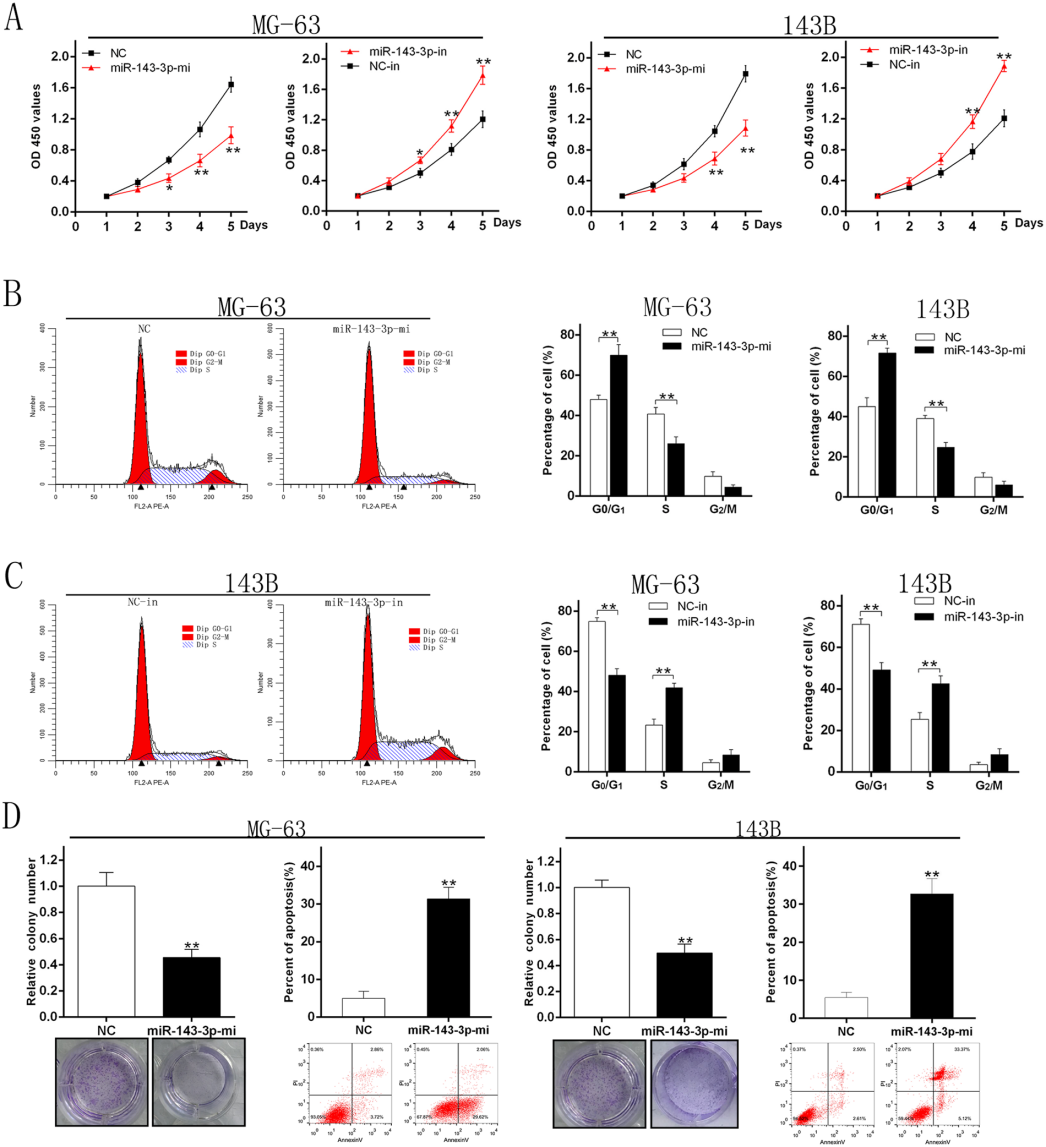
miR-143-3p inhibits proliferation of OS cells. (**A**) MG-63 and 143B cell lines were transfected with miR-143-3p mimics and miR-NC and the cell viability determined with the CCK-8 assay. (**B**) The results of flow cytometry analysis of MG-63 and 143B cell lines after transfected with miR-144 mimics and miR-NC. (**C**) The results of flow cytometry analysis of 143B cell lines after transfected with miR-143-3p-in and NC-in. **(D)** Results of colony formation and apoptosis cells of MG-63and 143B cell lines transfected with miR-143-3p mimics and miR-NC.

To determine the effect of miR-143-3p on proliferation of OS cells, we transfected miR-NC, miR-143-3p mimic, miR-NC-inhibitor and miR-143-3p inhibitor into the two cell lines. The CCK-8 assay was used to measure cell proliferation. The results showed that the proliferation of MG-63 and 143B cells after transfection with miR-143-3p mimic was significantly inhibited compared with the miR-NC group. Meanwhile transfection of the miR-NC-inhibitor and miR-143-3p inhibitor significantly increased the proliferation of MG-63 and 143B cell lines compared to the miR-NC-inhibitor group (Fig. 2A). These results indicated that miR-143-3p could inhibit the growth of OS cells. The results of cell cycle analysis showed that up-regulation of miR143-3p increases the number of cells in G1 phase and synchronously decreases the number in S phase, whereas inhibition of miR-143-3p decreases the number of cells in G1 phase and increases the number in S phase (Fig. 2B and C). All these results indicated that miR-143-3p could inhibit proliferation by blocking the G1/S phase transition. Moreover, the apoptosis rate was significantly higher in the two cell lines over-expressing miR-143-3p (Fig. 2D).

### miR-143-3p expression inhibits migration and invasion of OS cells *in vitro*

**Figure 3:**
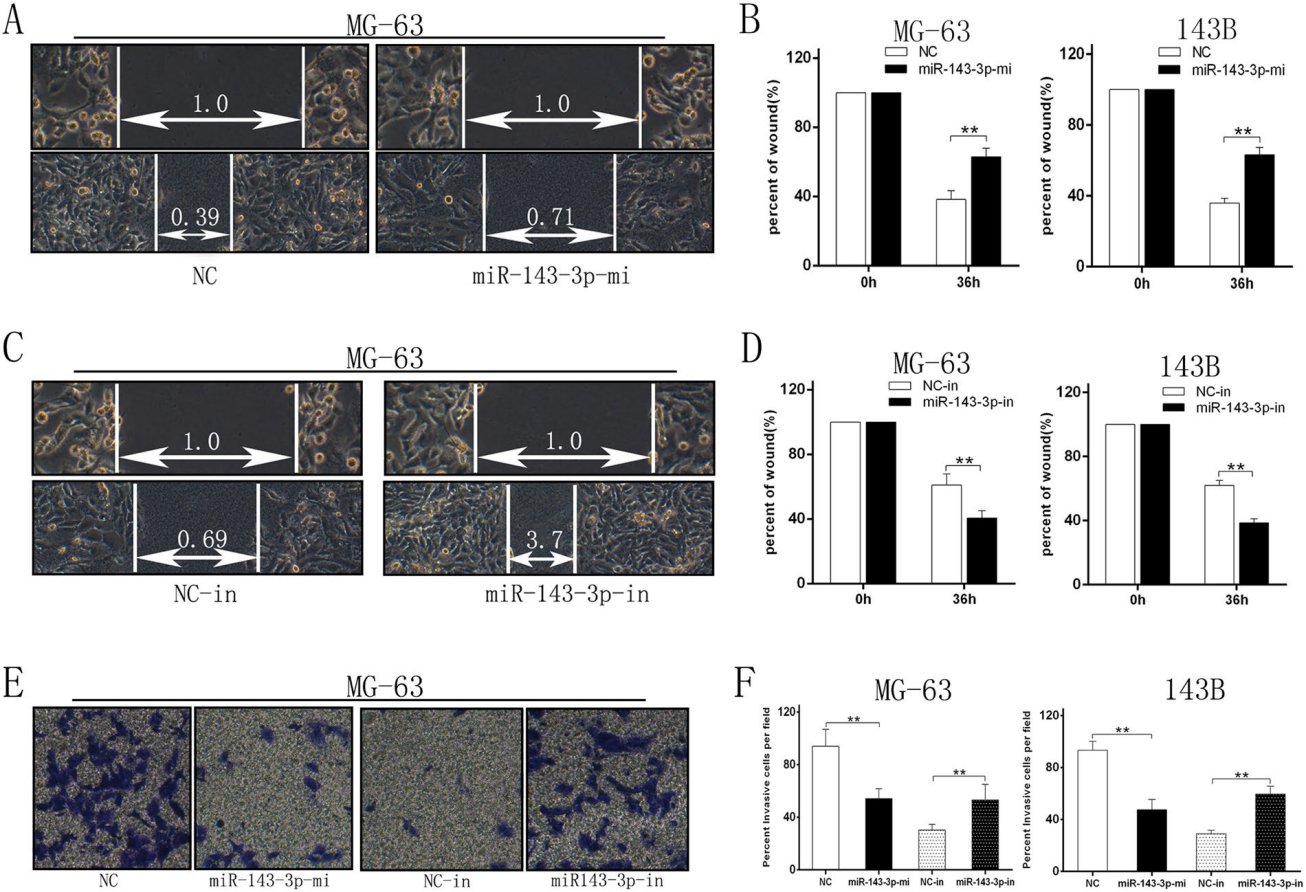
miR-143-3p expression inhibits migration and invasion of OS cells *in vitro*. (**A**,**B)** Wound-closure assay for MG-63 and 143B cell lines transfected with miR-144 mimics and miR-NC. **(C**,**D)** Wound-closure assay for MG-63 and 143B cell lines transfected with miR-143-3p-in and NC-in. **(E)** Images of the Transwell invasion assay of MG-63 cell line transfected with miR-144 mimics, miR-NC, miR-143-3p-in and NC-in. (**F**) Quantification of Transwell invasion assay for MG-63 and143B cell lines.

To further explore the function of miR-143-3p in migration and invasion of OS, we transfected the miR-NC, miR-143-3p mimic, miR-NC-inhibitor and miR-143-3p inhibitor into MG-63 and 143B cell lines. The wound healing assay showed that after up-regulation of miR-143-3p expression, migration and invasion of tumor cells was decreased compared with the control group (Fig. 3A and B). In contrast down-regulating miR-143-3p expression would lead to increased migration compared with the control group (Fig. 3C and D). The invasion assay showed that the invasive ability of the two cell lines was decreased in the miR-143-3p mimic group compared to the control group, while inhibiting miR-143-3p expression increased the cell’s invasive ability compared to the control group (Fig. 3E and F). This shows that miR-143-3p can inhibit cell migration *in vitro*.

### miR-143-3p inhibit tumor growth and metastasis *in vivo*

**Figure 4:**
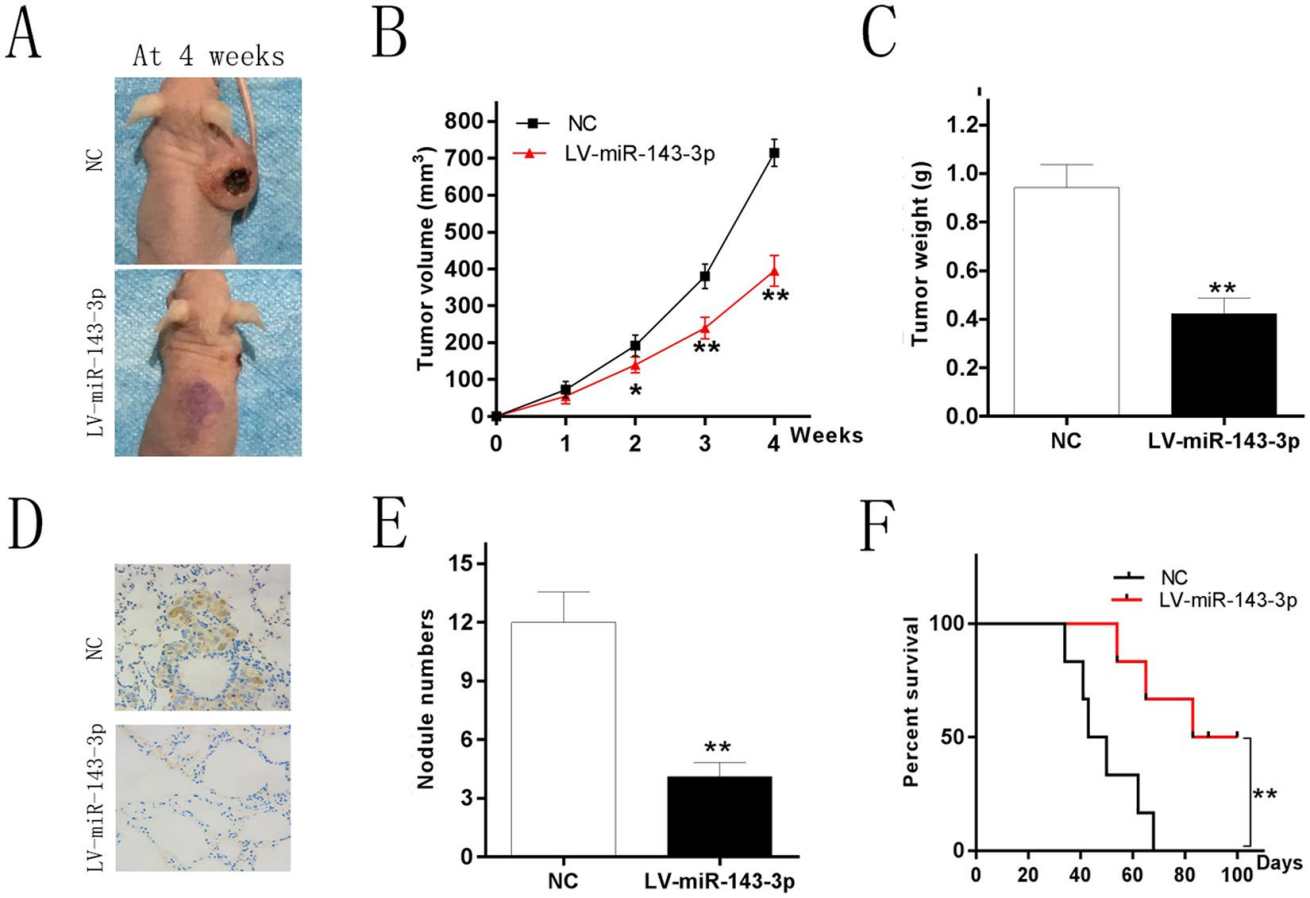
miR-143-3p inhibit tumor growth and metastasis *in vivo*. **(A)** Photographs of tumors (**B**) Curve of tumor volume growth for the nude mice. (**C**) Tumor of weight. (**D**) KI-67 staining section of lung tissue. (**E**) Number of lung metastatic nodules of each group. **(F**) Survival curve of the two groups mice injected the cell line respectively which could stably express the miR-143-3p mimics and miR-NC.

Based on the foregoing results, we conducted animal experiments to determine the function of miR-143-3p *in vivo*. The two cell lines which could stably express the miR-143-3p mimic and miR-NC were injected subcutaneously into nude mice and the animals were monitored for tumor growth for 4 weeks. The results showed that tumors which expressed miR-143-3p mimic were significantly smaller in volume and weight compared to the control group (Fig. 4A and B). Lung tissue of nude mice was also removed to determine the effects of miR-143-3p on tumor metastasis. We found that the number of lung metastasis nodules was dramatically decreased in the miR-143-3p group compared with the control group (Fig. 4C). This indicates that miR-143-3p could inhibit tumor growth and metastasis *in vivo*. Another group of mice was monitored for 100 days to construct a survival curve. The results showed that the survival time and rate of mice in high miR143-3p expression group were significantly higher than in the control group (Fig. 4D).

### miR-143-3p directly targets FOSL2

**Figure 5:**
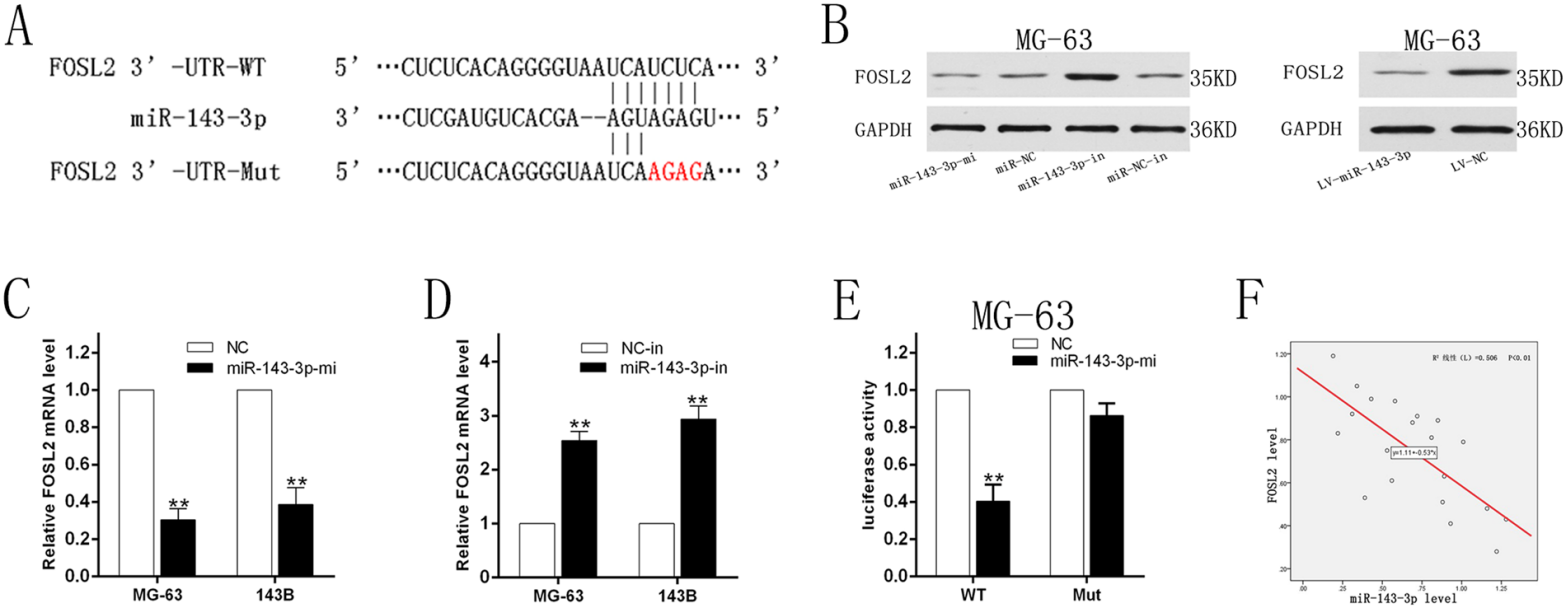
miR-143-3p directly targets FOSL2. **(A**) The sequences of the putative miR-143-3p binding sites in wild type and mutant (red) FOSL2-3′UTR. **(B)** Determination of FOSL2 protein in MG-63 cell after transfection with miR-144 mimics, inhibitors and miR-NC, NC-in or miR-144 lentivirus infection. (**C**) Determination of FOSL2 mRNA in MG-63 and 143B cell lines after transfected with miR-144 mimics and miR-NC. (**D**) FOSL2 mRNA in MG-63 and 143B cell lines after transfected with miR-143-3p-in and NC-in. (**E**) The relative luciferase activity of luciferase reports with wild type or mutant FOSL2-3′UTR were determined in MG-63 cell line, which were transfected with the miR-143-3p mimics or miR-NC. Statistical significance was observed between the wild type and the mutant groups. **(F)** Spearman’s correlation analysis between miR-143-3p expression and FOSL2 mRNA level.

We used the TargetScan, MiRanda, and PicTar databases to retrieve and draw the result of the search and identified FOSL2 as a possible target gene for miR-143-3p (Fig. 5A). To further verify its accuracy, we transfected the miR-143-3p mimic and miR-NC into MG-63 and 143B cell lines. Through this experiment we found that increasing the expression of miR-143-3p in cells significantly decreased the expression of FOSL2 compared to the miR-NC group (Fig. 5B and C). Then we transfected the miR-143-3p inhibitor and miR-NC-inhibitor into the two cell lines. The results showed that the expression of FOSL2 significantly increased compared to the control group (Fig. 5B and D). A luciferase reporter gene test showed that miR-143-3p mimic significantly decreased the activity of FOSL2 3′-UTR compared with the mutant group (Fig. 5E). Spearman’s correlation analysis was carried out using SPSS 22.0 software (Fig. 5F). These data suggest that FOSL2 is the target gene of miR-143-3p and negatively correlated with miR-143-3p.

### FOSL2 downregulation is a critical step in regulation of OS properties by miR-143-3p

**Figure 6:**
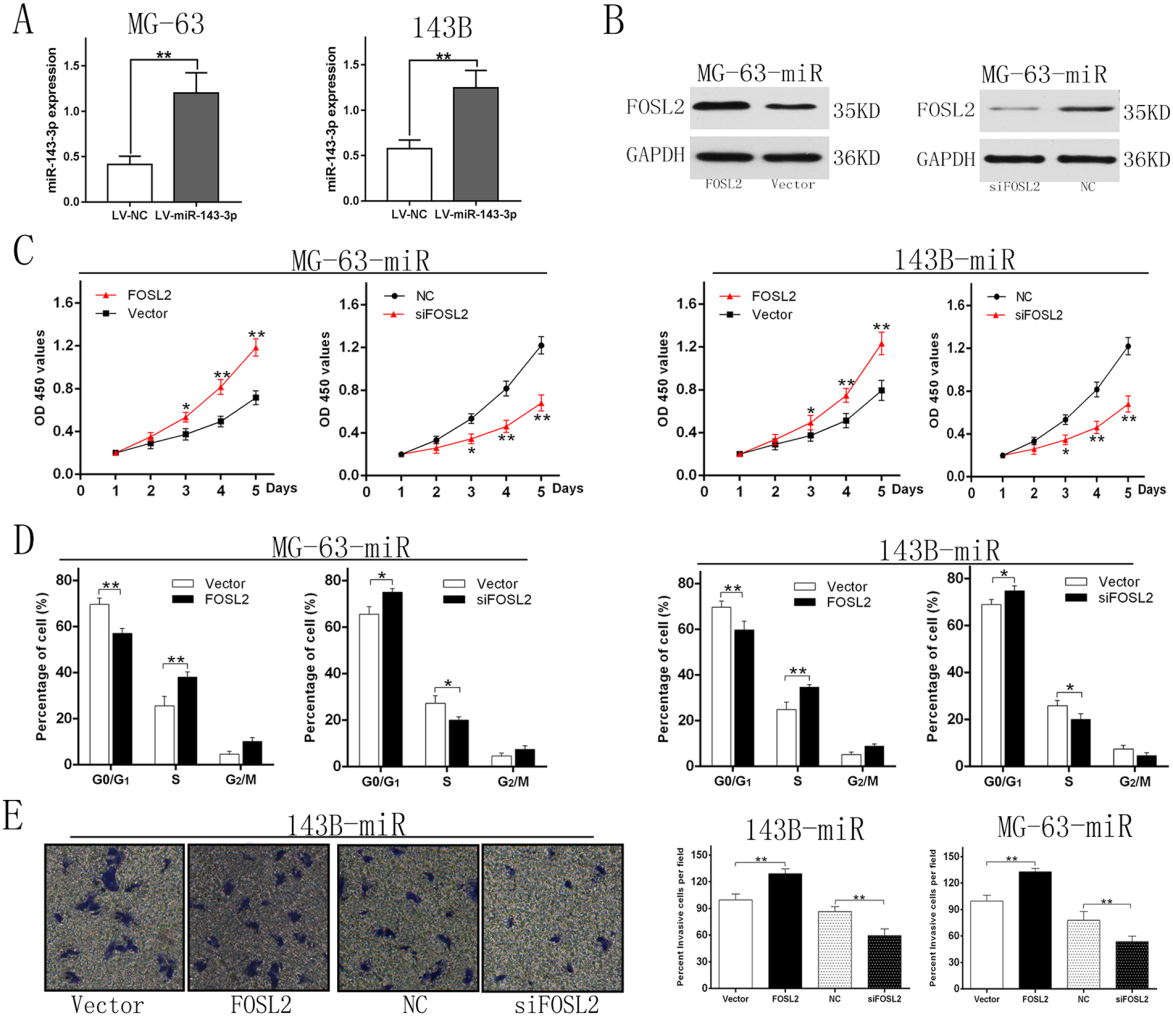
FOSL2 downregulation is a critical step in regulation of OS properties by miR-143-3p. (**A**) The miR-143-3p expression after transfected with lentivirus vectors. (**B**) (left) MG-63-miR cell (stably express miR-143-3p) were transfected with empty pcDNA3.1 vector, FOSL2-containing plasmid; (right) MG-63-miR cell were transfected with siRNA against FOSL2. (**C**) Cell viability of the two cell lines were measured by CCK-8 assay. The results showed that cell viability of ectopic FOSL2 group was higher than the control group. And the cell viability of siFOSL2 group was lower than NC group. (**D**) Cell cycle distribution were measured by flow cytometry analysis. The effects of G1/S phase blocking was weakened by ectopic FOSL2 and enhanced by siFOSL2. (**E**) Image and quantification of cell invasion ability were measured by Transwell assay.

Based on the foregoing results, we speculated whether miR-143-3p affected OS characteristics through regulating FOSL2 expression. To verify our conjecture, we transfected construct containing FOSL2 without the 3′-UTR into the cell lines which could stably express the miR-143-3p mimic (Fig. 6A and B). The results showed that the ectopic expression of FOSL2 could significant reversed the inhibition caused by miR-143-3p. Meanwhile silencing FOSL2 by specific siRNA led to enhanced the inhibition caused by miR-143-3p (Fig. 6C–E). Therefore, FOSL2 down-regulation is a critical step in regulation of OS properties by miR-143-3p.

## Discussion

OS is a common malignant tumor of bone often occurring in children and adolescents under the age of 20 years^14^. Some reports suggest that OS may originate from mesenchymal cells as it is prone to local invasion and easily undergoes distant metastasis resulting in a poor prognosis and high mortality^15^. The metastasis of OS is always one of the main factors that influences the survival rate of OS patients^3,16^. Under current treatment, the 5-year-survival-rate of patients without metastasis is 60–70%, while the 3-year-survival-rate for patients with distant metastasis is less than 30%^17,18^. In addition, the lung is the main metastasis site of OS and lung metastasis is the most important and frequent cause of death^19–21^. Recent studies have found that multiple miRNAs are expressed abnormally in OS cells and play roles as oncogenes and tumor suppressor genes in the occurrence and development of OS^10,12^. As the related literature reports, miRNAs participate widely in tumor development and inhibit tumor occurrence and development through there effect on target genes in tumor; for example miR-143-3p inhibits prostate cancer invasion and metastasis by targeting gene MMP13^22,23^, and miR-497 and miR-195 inhibit the invasion and metastasis of breast cancer by targeting the protein Raf-1 and Ccnd1^24,25^.

The down-regulation of miR-143 expression has also been reported in several human cancers, including colorectal cancer^23^, prostate cancer^26^, cervical cancer^27^, ovarian cancer^28^ and B-cell lymphoma^29^. Thus, it is considered that miR-143 is a tumor-suppressor miRNA. We analyzed expression of miR-143-3p in OS cells and adjacent normal tissues and found that the expression of miR-143-3p in tumor tissues decreased significantly compared with adjacent normal tissues, similar to previous report. This indicates that the decrease of miR-143-3p expression may play a role in the occurrence and development of OS. Low expression of miR-143-3p was linked to distant metastasis and poor prognosis in OS patients. This may provide a new way to predict the prognosis for OS patients.

In general, miRNAs play a regulatory role by directly inhibiting target genes^30–32^. To further study the mechanism of miR-143-3p in the occurrence and development of OS, we infected MG-63 and 143B cell lines using a specific virus. The effect of miR-143-3p on cell proliferation and apoptosis is controversial. Fan *et al*. reported that miR-143-3p could inhibit tumor cell proliferation and promote apoptosis^23^. Meanwhile Osaki *et al*. showed that miR-143-3p has no effect on the proliferation or apoptosis of OS^33^. Akao *et al*. reported that the target gene of miR-143 was determined to be ERK5/MAPK7, the up-regulation of which leads to cell growth via activation of c-Myc, in Raji cells, a human B-cell lymphoma cell line^34^. Recently, another publication showed that miR-143 suppressed cell proliferation by inhibiting KRAS translation in human colorectal cancer^35,36^. Our study found that the apoptosis rate of tumor cells increased significantly after up-regulation of miR-143-3p expression, indicating that miR-143-3p is involved in the process of apoptosis. Thus the decreased expression of miR-143-3p may lead to the development of tumors.

Cell migration experiments showed that in the MG-63 and 143B cell lines, cell migration ability increased significantly after knockdown of miR-143-3p expression, while expression increased after inhibition of miR-143-3p function. All the results showed that miR-143-3p was closely related with OS which was prone to migratory and invasive properties. These findings provide a new theoretical basis for further treatment of OS. Not only that, we can also diagnose and predict OS by detecting the expression of miR-143-3p in tissues, serum or other specimens.

The results of *in vitro* experiments showed that miR-143-3p-mimics inhibited the invasion and metastasis of OS cell lines. To investigate the detailed mechanism, we identified the target gene of miR-143-3p through searching TargetScan, MiRanda and PicTar databases and confirming the results using the Luciferase reporter gene test. FOSL2 belongs to the activator protein 1 (activator protein, l, APl) transcription factor family^37,38^. FOSL2 is widespread in human tissues and plays an important role in mammalian growth, reproductive traits, and immune response^39,40^. The expression products of FOSL2 are related to cell adhesion, movement, invasion, metastasis and cell growth^37^. Studies have shown that over-expression of FOSL2 is associated with a higher invasive association with breast cancer and involved in breast cancer progression^39^. In addition, FOSL2 also plays a role in the TGF- beta signaling pathway, and is essential for TGF- beta1 to induce cell migration.

**Figure 7:**
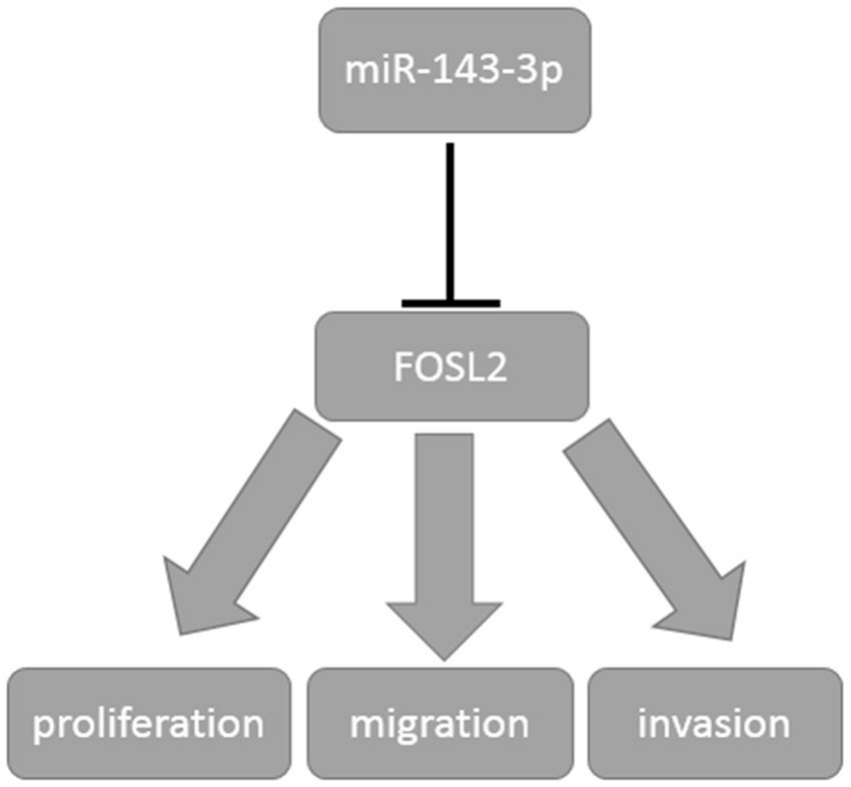
Schematic diagram of the regulation of miR143-3p to FOSL2. miR-143-3p could inhibit the proliferation, migration and invasion of OS by regulating the expression of FOSL2 negatively.

Finally, our results showed that when the expression level of miR-143-3p in cells was increased, the expression level of FOSL2 decreased significantly. In contrast, inhibiting miR-143-3p expression led to a dramatic increase in the expression level of FOSL2. We also found that FOSL2 expression could significantiy reverse the inhibition caused by miR-143-3p. Furthermore, silencing of FOSL2 by specific siRNA enhanced the inhibitory effects caused by miR-143-3p. These data suggest that down-regulation of FOSL2 is a critical step in regulation of OS properties and the expression of miR-143-3p can inhibit the proliferation, migration and invasion of OS by reducing the expression of FOSL2 (Fig. 7).

In conclusion, the decrease of miR-143-3p expression in OS cells is closely related to the occurrence and development of OS. We have shown that miR-143-3p directly targets FOSL2 and affects the OS characteristics through down-regulation of FOSL2 expression. This provides us with a new target for the treatment of OS, and is deserving of further study.

## Materials and Methods

### OS tissue samples

The study was approved by the Ethics Committee of Renmin Hospital of Wuhan University. Written informed consent was obtained from the patients and their parents/guardians prior to tissue collection. All procedures were conducted in accordance with guidelines provided by the Ethics Committees and Institutional Review Boards. In brief, osteosarcoma tissue specimens (n = 33) were gathered from 2013 to 2017 at Renmin Hospital of Wuhan University and 20 adjacent normal tissues were collected at the time of operation simultaneously. The relevant clinical information was collected from patients with prior informed written consent. All osteosarcoma specimens were frozen in liquid nitrogen and then cryopreserved in −80 °C. Tumor stage and grade were classified according to the Enneking staging system.

### Cell culture

Three cell lines: hFOB 1.19 (human fetal osteoblastic cell line), MG-63 and 143B were purchased from the Institute of Cell Bank for Biological Sciences (Shanghai, China). MG-63 and 143B were cultured in DMEM (Hyclone, Utah, USA) containing 10% FBS (Gibco, Australia) and 1% antibiotics (penicillin 100 U/ml, streptomycin 100 μg/ml, Hyclone, Utah, USA) at 37 °C with 5% CO_2_ and 100% humidity. hFOB1.19 cell line was maintained in DMEM/F-12 (Hyclone, Utah, USA) supplement 10% FBS (Gibco, Australia) and geneticin (400 μg/ml, Sigma, USA) at 34 °C in a saturated humidified incubator. Trypan blue staining was used to determine the Cell viability.

### Quantitative real-time PCR

Total RNA was extracted from clinical specimens and OS cell lines and hFOB1.19 cells using RNeasy Plus Mini Kit (Life Technology, US) according to the manufacturer’s instructions. The RNA concentration and purity was determined using an ND-1000 spectrophotometer and stored at −80 °C. Then, cDNA of miRNA was synthesized from 100 ng of total RNA using TaqManH MicroRNA Reverse Transcription (ABI, CA) and cDNA of RNA was synthesized from TaqMan 1 μg of total RNA using Reverse Transcription Reagents (ABI, CA). MiRNAs were reverse-transcribed using specific RT primers; mRNAs were also reverse-transcribed by using random hexamers. The primer sequences used were as follows: FOSL2 forward and reverse, 5′-GAGAGGAACAAGCTGGCTGC-3′ and 5′-GCTTCTCCTTCTCCTTCTGC-3′, respectively; miR-143-3p forward and reverse, 5′-GGGGTGAGATGAAGCACTG-3′ and 5′-CAGTGCGTGTCGTGGAGT-3′, respectively; GAPDH forward and reverse, 5′-ACTTTGGTATCGTGGAAGGACTCAT-3′ and 5′-GTTTTTCTAGACGGCAGGTCAGG-3′, respectively; U6 snRNA forward and reverse, 5′-GCTCGGCAGCACATATACTAAAAT-3′ and 5′-CGCTTCACGAATTTGCGTGTCAT-3′, respectively. Real-time quantitative PCR (qRT-PCR) was performed using SYBR Premix ExTaqTM (Invitrogen, US). GAPDH (for mRNAs) or U6 small nuclear RNA (for miRNAs) was used as an internal control. The relative amount of mRNA or miRNA was calculated according to the comparative threshold cycle value (2^−ΔΔCt^) method. All operations were repeated at least three times.

### Lentivirus infection and oligonucleotide transfection

miR-143-3p mimics (5′-UGAGAUGAAGCACUGUAGCUC-3′), complementary chain (5′-GAGCUACAGUGCUUCAUCUCA-3, miR-143-3p inhibitor (5′-GAGCUACAGUGCUUCAUCUCA-3′) were artificially synthesized from GeneChem Company (Shanghai, China). siRNA for FOSL2 was obtained from GeneChem Company (Shanghai, China). pcDNA3.1 (+)-FOSL2 plasmid was consisted of FOSL2 coding sequences and pcDNA3.1 (+). Transfection of oligonucleotide and plasmids was performed using Lipofectamine 2000 reagent (Invitrogen, USA). The lentivirus vectors which could express miR-143-3p and control vector were obtained from GeneChem Company (Shanghai, China). The lentiviral vector and its packaging vectors were co-transfected into 293T packaging cells to obtain indicated lentivirus. Target cells were infected with fresh lentivirus-containing medium. Stably transfected cells (LV- miR-143-3p) were selected by antibiotic resistance (puromycin, 2 μg/mL) for two weeks.

### Luciferase assay

We used the pGL3-reporter luciferase vector to construct the pGL3-FOSL2 3′UTR or pGL3-FOSL2 3′UTR-mut vectors. Vectors expressing miR-143-3p were co-transfected to examine the Luciferase activity. After transfection of 48 h, the Luciferase activity were measured using Dual luciferase reporter assay system (Promega, USA). The ratio of Firefly to Renilla luciferase activity was used as the relative activity of luciferase. All operations were repeated at least three times.

### Cell proliferation, colony formation, cell cycle and apoptosis assays

OS cells with different processing methods in different experimental groups were digested and seeded in 96 well plates. Using the CCK-8 assay to detect the cell growth and drawing cell growth curve according to the protocol of the CCK-8 kit (Dojindo, Japan). Cell cycle and apoptosis was analyzed by flow cytometer (BD, USA). For cell cycle analysis, cells were harvested and treated with RNase A (300 μg/ml, Sigma-Aldrich, USA), and then stained with propidium iodide (PI) (10 μg/ml, Sigma-Aldrich, USA). The stained nuclei were measured by flow cytometer. We used Annexin V-FITC/PI kit (BD, USA) to analysis and quantify the cell apoptotic. According to the manufacturer’s protocol. Cells were washed and resuspended at a concentration of ~1 × 10^6^ cells/ml. After treated with Annexin V-FITC and PI solution, cells were stained by Annexin V-FITC/PI for 10~15 min, away from light. Flowjo software was used to determine the apoptosis rate.

### *In vitro* wound-closure assay

OS cell lines suspension (5 × 10^5^/ml) was seeded on 6-well plates and incubated overnight at 37 °C, 5%CO_2_ incubator. The experiment was divided into 4 groups: miR-143-3p-mi group, miR-143-3p-NC group, miR-143-3p-in group and miR-143-3p-in-NC group. After cell attachment, transfection was performed. All operations were performed without RNA enzymes. 500 μl transfection solution was added to each well. Using a 200 μl pipette tip drew a straight line vertically at the center of each well, and the healing of 0 and 36 h was recorded under the microscope and the healing area of the wound was recorded. Each experiment was repeated three times.

### Transwell invasion assays

Cell invasion assays was performed using the Transwell membranes (Corning Inc., New York, NY, USA). Transwell invasion assays were performed according to the manufacturer’s protocol. Briefly, cells were seeded in the upper chamber of the Transwell invasion system and the low chambers were filled with culture medium as chemoattractant and then stay for 48 hours. The cells that invaded into the lower chamber were stained and quantitated by counting under microscopy and taking pictures. The cell number was calculated by Image J software.

### Protein extraction and western blotting analysis

Total cell lysates were prepared with a detergent lysis buffer. Western blots were performed as instructions. Anti-FOSL2 and anti-GAPDH (CST, Beverly, MA, USA) were used as first antibody. After washing with PBST buffer, membranes were incubated with secondary antibodies for 2 h. Finally, an enhanced chemiluminescence substrate were used to treat the membranes. GAPDH was used as the loading control.

### Mouse models

Four-week-old BALB/c nude mice were obtained from Beijing Weitong Lihua Experimental Animal Technology Co. Ltd. and maintained at Center for Animal Experiment, Renmin Hospital of Wuhan University. The animal experiments were approved by the Institutional Review Board of the Renmin Hospital of Wuhan University and were undertaken in accordance with the National Institutes of Health Guide for the Care and Use of Laboratory Animals. 5 × 10^6^ cells /0.2 ml of 143B-vector or 143B-miR-143-3p cells were injected subcutaneously into the flanks of mice. Each group was randomly assigned to 6 nude mice. The tumor was measured with a caliper once a week for up to 4 weeks. To compare the survival of different mice, 5 × 10^5^ cells /0.2 ml were inoculated subcutaneously and mice survival were also recorded up to 100 days. The tumor volume (V) (mm^3^) was calculated as the formula: V = (W^2^ × L)/2. And the curve of tumor growth was depicted 4 weeks after inoculation.

### Statistical analysis

SPSS 22.0 software was used for statistical analysis. Independent sample Student’s t-test was used if the quantitative data between groups show normal distribution. If not consistent with the normal distribution, using the Wilcoxon-Mann-Whitney test. The survival curve was calculated using the Kaplan-Meier method and statistical significance was determined by the Log-Rank test. P < 0.05 indicated that the difference was statistically significant.
